# 3D Ultrasound Measurements Are Highly Sensitive to Monitor Formation and Progression of Abdominal Aortic Aneurysms in Mouse Models

**DOI:** 10.3389/fcvm.2022.944180

**Published:** 2022-07-12

**Authors:** Nahla Ibrahim, Sonja Bleichert, Johannes Klopf, Gabriel Kurzreiter, Viktoria Knöbl, Hubert Hayden, Albert Busch, Alexander Stiglbauer-Tscholakoff, Wolf Eilenberg, Christoph Neumayer, Marc A. Bailey, Christine Brostjan

**Affiliations:** ^1^Division of Vascular Surgery, Department of General Surgery, Medical University of Vienna, Vienna General Hospital, Vienna, Austria; ^2^Department for Visceral, Thoracic and Vascular Surgery, Technical University of Dresden, University Hospital Carl-Gustav Carus, Dresden, Germany; ^3^Division of Cardiovascular and Interventional Radiology, Division of Molecular and Gender Imaging, Department of Biomedical Imaging and Image Guided Therapy, Medical University of Vienna, Vienna General Hospital, Vienna, Austria; ^4^School of Medicine, Leeds Institute for Cardiovascular and Metabolic Medicine, University of Leeds, Leeds, United Kingdom; ^5^Leeds Vascular Institute, Leeds General Infirmary, Leeds, United Kingdom

**Keywords:** 3D ultrasound, abdominal aortic aneurysm, aortic volume, maximum aortic diameter, cardiovascular imaging, murine models

## Abstract

**Background:**

Available mouse models for abdominal aortic aneurysms (AAAs) differ substantially in the applied triggers, associated pathomechanisms and rate of vessel expansion. While maximum aortic diameter (determined after aneurysm excision or by 2D ultrasound) is commonly applied to document aneurysm development, we evaluated the sensitivity and reproducibility of 3D ultrasound to monitor aneurysm growth in four distinct mouse models of AAA.

**Methods:**

The models included angiotensin-II infusion in ApoE deficient mice, topical elastase application on aortas in C57BL/6J mice (with or without oral administration of β-aminoproprionitrile) and intraluminal elastase perfusion in C57BL/6J mice. AAA development was monitored using semi-automated 3D ultrasound for aortic volume calculation over 12 mm length and assessment of maximum aortic diameter.

**Results:**

While the models differed substantially in the time course of aneurysm development, 3D ultrasound measurements (volume and diameter) proved highly reproducible with concordance correlation coefficients > 0.93 and variations below 9% between two independent observers. Except for the elastase perfusion model where aorta expansion was lowest and best detected by diameter increase, all other models showed high sensitivity of absolute volume and diameter measurements in monitoring AAA formation and progression by 3D ultrasound. When compared to standard 2D ultrasound, the 3D derived parameters generally reached the highest effect size.

**Conclusion:**

This study has yielded novel information on the robustness and limitations of semi-automated 3D ultrasound analysis and provided the first direct comparison of aortic volume increase over time in four widely applied mouse models of AAA. While 3D ultrasound generally proved highly sensitive in detecting early AAA formation, the 3D based volume analysis was found inferior to maximum diameter assessment in the elastase perfusion model where the extent of inflicted local injury is determined by individual anatomical features.

## Introduction

An aneurysm is considered a focal, degenerative and progressive dilatation of the vessel wall, with the risk of vessel rupture (1). Abdominal aortic aneurysms (AAAs) occur at a worldwide prevalence of about 5% with substantial regional variation (2–4). Risk factors include smoking, hypertension, advanced age, male sex, and genetic susceptibility (5, 6). A typical feature is the common presence of an intraluminal thrombus (ILT) in 75% of clinically evident AAAs (7). While pharmacological treatments have been evaluated, the only current curative option is surgical repair (8, 9). Therefore, the need of preclinical models to understand the pathobiology in early aneurysm disease and assess the potential of novel drug therapy is evident (10). Multiple models of small- as well as large-animal AAAs are available, with murine models being the most widely applied experimental systems (11, 12).

The porcine pancreatic elastase (PPE) model was first described in wildtype 129/SvJ mice by Pyo et al. (13). The abdominal aorta was isolated *in vivo* from the adjacent inferior *vena cava*, ligated and cannulated for perfusion with saline containing 0.4 U/ml type I PPE. The authors reported a 74 ± 5% dilatation of the abdominal aorta during the 5 min period of elastase perfusion, but no further increase in aortic diameter up to 7 days after surgery. After 14 days the AAA development was apparent, at 91% incidence and a mean aortic diameter of 134 ± 8% (13).

The angiotensin II (AngII) model was introduced by Daugherty et al. (14). In this study, AngII was infused at 1,000 ng/kg/min via subcutaneously implanted osmotic minipumps into ApoE KO mice, 6 months of age, for 28 days at which point AAAs had formed in 33% of animals. A subsequent study showed three prominent features of this model: a high vessel rupture rate within the first 2 weeks, frequently concurring thoracic and abdominal aneurysms as well as aorta dissections with intramural thrombi (14, 15).

A modification of the PPE model was first described by Bhamidipati et al. (16), where 8–10 week old male C57BL/6 mice received peri-adventitial application of porcine pancreatic elastase (ePPE): The dissected abdominal aorta was immersed in 10 μl 100% elastase. Initially, the aorta dilated in surgery by about 30–40%, followed by continuous aneurysmal expansion over 2 weeks. On day 14, the group reported 82 ± 15% increase in aortic diameter, with a 60% incidence rate.

In 2017, the group of Lu et al. (17) aimed to evolve the ePPE technique and establish a chronic model of AAA with beta-aminopropionitrile (BAPN) which inhibits elastin and collagen crosslinking. Male C57BL/6 mice at 8 weeks of age received 0.2% BAPN in drinking water starting 2 days before surgery and continuing until the end of the study. Mice having BAPN in addition to the ePPE procedure showed a higher rate of aneurysm formation (93% incidence) than mice receiving no BAPN (65%). Pronounced aneurysm progression was observed on days 21 (222 ± 37%), 28 (286 ± 79%), and 100 (801 ± 160%), including considerable thrombus formation (54%) and rupture (31%) at the advanced AAA stage (17).

Preclinical monitoring of AAA formation and progression can be performed through various imaging techniques, with ultrasound (US) being the most frequently applied method (18). One of the main advantages of using US imaging is the lack of ionizing radiation. Preclinical systems use higher frequencies (15–40 MHz) than clinical systems, resulting in the benefit of higher spatial resolution at the cost of decreased imaging depth (19). The mode most commonly used is B-mode or brightness-mode, in which 2D cross-sectional images are acquired. Waduud et al. have described the method of using 3D ultrasound for the evaluation of AAA development in the murine ePPE model (20). With the help of an automatic motor and an MS-550D probe at 40 MHz frequency, transverse imaging was performed and the 3D volume was calculated from 157 serial images recorded along a 12 mm abdominal aortic segment.

We thus hypothesized that a sensitive imaging method as developed by Waduud et al. (20) may be suited to detect AAA formation at early time points in small-animal models and monitor their further development. This would offer the possibility to preferably test novel treatments on formed aneurysms and thus address the clinical demand to limit progression of established disease. While Waduud et al. (20) focused on the ePPE model as proof-of-principle, this study thus aimed to,

(1) Evaluate the time course of aneurysm formation and development in four AAA mouse models (AngII, PPE, ePPE, and ePPE + BAPN) by 3D ultrasound.
(2) Assess the robustness and interobserver variability of 3D volume vs. aortic diameter measurements in these models.
(3) Compare the sensitivity of aortic volume and maximum diameter to detect significant aneurysm growth at early time points for potential stratification into treatment groups.

## Materials and Methods

### Ethical Approval

All animal experiments were approved by the local ethics committee and the Austrian Ministry of Science (BMWFW-66.009/0355-WF/V/3b/2016 and 0248-WF/V/3b/2017 for the AngII and PPE models, 2020-0.547.895 for the ePPE ± BAPN models), conforming to the European Directive 2010/63/EU on the protection of animals used for scientific purposes and the Austrian Animal Experiment Act 2012.

For pain management, the animals were injected subcutaneously with 0.05 mg/kg buprenorphine before surgeries. Until the 3rd postoperative day, mice were given 7.5 mg piritramide and 20 ml 5% glucose in 200 ml drinking water (0.9% NaCl).

### Murine Abdominal Aortic Aneurysm Models

**FIGURE 1:**
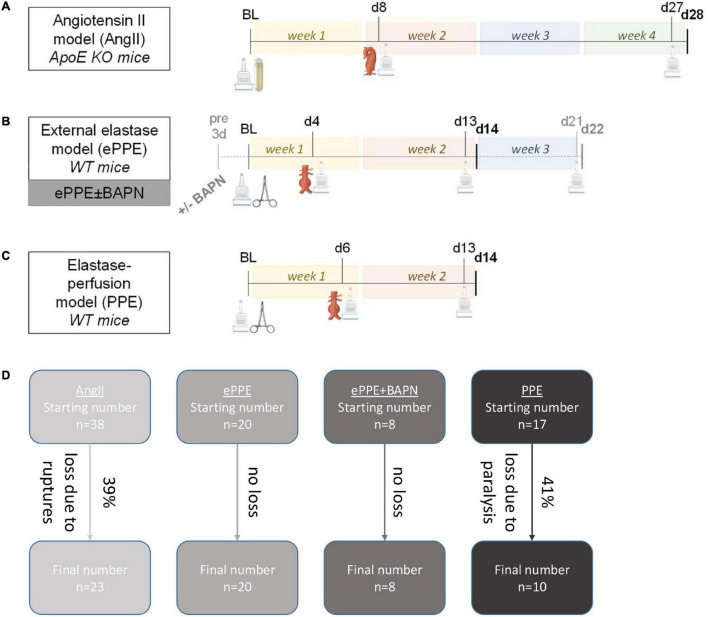
Experimental design of AAA mouse models. Experimental steps and time lines are illustrated for the **(A)** AngII model, **(B)** ePPE ± BAPN models and **(C)** PPE model. Time points of 3D US analysis are indicated by the scanner icon, while interventions at baseline to trigger AAA development are illustrated by osmotic pump for the AngII model or by surgical clamp for the elastase-based models. Formed aneurysms were either of suprarenal (AngII) or infrarenal (elastase models) localization. For the ePPE + BAPN model (gray lettering), BAPN was administered from 3 days before surgical intervention until day 22 as opposed to the ePPE model without BAPN which was terminated on day 14. **(D)** The flow chart illustrates starting numbers and animal losses in the four mouse models resulting in the final number of mice included in the follow-up time points.

While the technical procedures of the 4 murine AAA models are specified below, a graphical summary of the timelines and experimental steps of the individual models is given in Figures 1A–C. Please note that all mice included in this retrospective analysis represent control animals of other studies where phosphate-buffered saline injections started after the second ultrasound analysis (i.e., after initial aneurysm formation). All animals within each model were treated in the same manner; none of the mice received drug therapy.

### Angiotensin II Model

Male mice homozygous for the ApoE mutation (B6.129P2-Apoetm1Unc/J@Him), aged 11–15 weeks and kept on normal diet, received angiotensin II (AngII, Bachem, Bubendorf, Switzerland) at 1,000 ng/kg/min by subcutaneously implanted ALZET 2004 osmotic pumps (DURECT Corp., Cupertino, CA, United States) over 28 days, leading to formation of suprarenal AAAs. Measurements of AAA volume and diameter were conducted by ultrasound at baseline, on day 8 and on day 27.

### ePPE: External Porcine Pancreatic Elastase Model ± Beta-Aminopropionitrile

Male C57BL/6J mice aged 9–11 weeks, received topical peri-adventitial elastase application to the infrarenal aorta to induce AAA formation: The mice underwent open median laparotomy and the infrarenal portion of the aorta was separated from surrounding fat and connective tissue, creating a unilateral peri-aortic pouch where 10 μl of PPE (Sigma-Aldrich, St. Louis, MO, United States) at 7.6 mg/ml were applied for 5 min. The elastase was absorbed with a cotton swab and the abdomen flushed 3 times with saline before closure. Mice receiving BAPN (Sigma-Aldrich, St. Louis, MO, United States) were supplied at 2 g/l (0.2%) in drinking water from 3 days before AAA induction and over the entire course of the experiment. The aortic volume and diameter were monitored by 3D ultrasound at baseline, day 4 and d13 after elastase application in the ePPE model, and at baseline, day 4, day 14, and day d21 in the ePPE + BAPN model.

### Porcine Pancreatic Elastase Model

The infrarenal portion of the aorta was separated from surrounding fat and connective tissue in male C57BL/6J@Him mice aged 9–12 weeks. Side branches and the aorta itself were ligated with 6/0 silk ligatures (Ethicon, Cincinnati, OH, United States). The aorta was punctured and a catheter was inserted for perfusion of the infrarenal aorta with PPE at 2 U/ml (0.4 mg/ml) for 10 min. The aorta was then flushed with saline and closed with a single 10/0 suture (Ethicon), before opening of the ligatures and closure of the abdomen. Aortic volume and diameter were monitored by ultrasound at baseline, day 6 and day 13.

### 3D Ultrasound Recording

Prior to and during ultrasound sonography, mice were anesthetized with 1.8–2% isoflurane and 2 l/min O_2_. For ultrasound measurements of aneurysms, the following settings of the Vevo 2100 or Vevo 3100 Imaging System (FUJIFILM VisualSonics Inc., Toronto, ON, Canada) were applied: gain 30 dB, image depth 9 mm, image width 8.08 mm. Respiratory gating was set to 25% delay and a window of 50%; T1 50 ms were used for electrocardiogram trigger. After localization of the left renal artery, the MS-550D/MX-550D transmitter at 40 MHz was moved 6 mm cranially (for the AngII model) or 6 mm caudally (for the PPE and ePPE ± BAPN models) for an automated scan of the suprarenal or infrarenal aorta, respectively. In total, 157 imaging frames were generated over a scan distance of 12 mm with 0.076 mm step size. To ensure reliability of imaging, scans showing interference of any kind (for example derived from the transmitter, ultrasound gel, bowel movement, etc.) were repeated.

### Aortic Volume and Diameter Measurement Based on Ultrasound

Data was acquired as a video loop of the 157 frames and composed into a 3D cube with Vevo Lab 5.6.1 software. The 3D aneurysm reconstruction was based on software tools and required manual sketching of the aortic area (inner-to-inner wall) in axial plane at 0.75–1 mm intervals. The aortic volume was calculated in mm^3^ over the monitored distance of 12 mm. The maximum aortic diameter was also determined in axial plane (inner-to-inner wall) based on the 3D image acquisition. In case of thrombus development in dissected aortas (AngII model), thrombi were included in the aortic wall tracing. Both, aortic volume and maximum diameter were either expressed in absolute measurements (mm^3^ or mm, respectively) or were set in relation to baseline values and given in percent increase. The maximum aortic diameter was additionally assessed by a single recording in 2D ultrasound (transverse) B-mode or EKV-mode (electrocardiogram-gated kilohertz visualization), also inner-to-inner wall in axial plane.

### Reproducibility of Ultrasound Measurements

The scan of the 12 mm aortic stretch was performed only once, as it proceeds in an automated fashion after localization of the left renal artery. After export of the 157 images, two observers (PhD or MD students with a 2–4 year research engagement) independently conducted the analysis, i.e., the 3D aneurysm reconstruction as well as measurement of aortic volume and diameter. The team received prior training by the senior scientist who developed the 3D US protocol for AAA assessment (co-author Marc A. Bailey). The observers were blinded to each other’s results while conducting the reconstruction and measurements. Statistical analysis was independently confirmed by a third researcher (the corresponding author).

### External Measurement of Maximum Aortic Diameter

For *ex vivo* measurements of aortic diameter, mice were sacrificed via intraperitoneal injection of ketamine and xylazine and perfused with phosphate-buffered saline (without calcium and magnesium, PBS^–/–^), then 4% paraformaldehyde in PBS^–/–^ via the left ventricle. Images of the excised aorta were taken on a standardized measurement plate (Self-Healing Mat, Xcut West Design, Plymouth, United Kingdom) at 8x magnification through the Olympus SZ51 Stereo microscope (Olympus Corporation, Shinjuku City, Tokyo, Japan) and analyzed with ImageJ version 1.53j. After setting the scale to a 10 mm line on the measurement plate, *ex vivo* diameters were measured by manually drawing a line at the maximum diameter by two independent observers.

### Statistical Analysis

The study design was retrospective, i.e., 3D US data were acquired by the two independent observers while investigating pathomechanistic features of the four presented AAA models. Statistical analyses were performed using GraphPad Prism 8 (version 8.0.2) and SPSS (version 26.0). The main parameters were absolute aortic volume and diameter as well as relative aortic volume and diameter, normalized to the baseline value of 100%. Results displayed over time are averaged values of the two independent observers and are shown as mean and standard deviation of comparably treated mice for each time point. The following statistical tests were applied to address the three major questions:

(1) To detect significant changes in AAA parameters over time for each mouse model, linear mixed effects modeling (LMEM) was performed specifying experimental animals as random effect and time as fixed effect, as either metric covariate or categorical factor when comparing individual time points.
(2) Reproducibility of AAA volume and diameter measurements was addressed by scatter plots, Lin’s concordance correlation coefficient and Spearman correlation coefficient between the data sets of the two independent observers. Furthermore, Bland-Altman plots with 95% limits of agreement were calculated.
(3) Correlation between volume and diameter measurements was depicted by scatter plots and Spearman coefficient. To compare aortic volume and maximum diameter in detecting aneurysm growth at early time points, Cohen’s standardized effect size was determined for the distinction between aortic size at baseline vs. first time point of AAA detection for all parameters and models.

## Results

Abdominal aortic aneurysms were induced in 38, 20, 8, and 17 mice based on the AngII, ePPE, ePPE + BAPN, or PPE experimental procedures (Figure 1D). While no animals were lost in the ePPE and the ePPE + BAPN groups, 15 mice (39%) experienced aortic ruptures within the first 10 days of AngII treatment and 7 mice were lost to postoperative paralysis (41%) after PPE perfusion. Hence, data from 23 AngII, 20 ePPE, 8 ePPE + BAPN, and 10 PPE mice were included in the follow-up time points. 3D US investigation was conducted at baseline, early and advanced stages of AAA development resulting in data sets of 84, 60, 32, and 37 aortic volume measurements for the AngII, ePPE, ePPE + BAPN, and PPE models. Of note, maximum diameter was additionally recorded for 77, 58, 32, and 37 data sets, respectively.

### 3D Ultrasound Analysis Yields Highly Reproducible Abdominal Aortic Aneurysm Measurements for All Mouse Models

**FIGURE 2:**
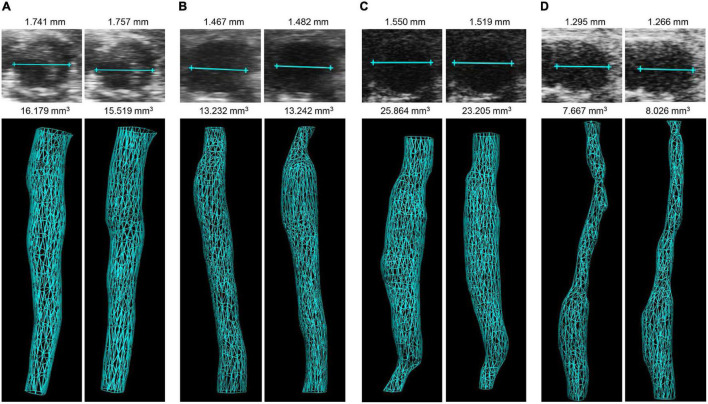
Illustration of 3D volume and maximum aortic diameter measurements in four AAA mouse models by two independent observers. Examples are given for diameter (upper panel) and 3D reconstructed volume (lower panel) in the **(A)** AngII model, **(B)** ePPE model, **(C)** ePPE + BAPN model, **(D)** PPE model, as assessed by observer 1 (left) and observer 2 (right). Of note, 3D vascular images represent inner lumen. In case of the AngII model thrombi were included in the aortic wall tracing.

Absolute values of both 3D reconstructed aortic volume and maximum aortic diameter (illustrated in Figure 2) were found to be highly reproducible between two independent observers in all four AAA mouse models, as calculated by the coefficient of variation (CV) listed in Table 1. While mean CVs consistently ranged below 9%, best reproducibility was achieved in the ePPE model (1.7% for aortic volume) and highest interobserver variation was recorded in the ePPE + BAPN model (8.5% for aortic volume). Comparably, intraobserver variation in the ePPE + BAPN model ranged only at 2.3% for volume and 0.9% for diameter. To set reproducibility measures in relation to sample size, Lin’s concordance correlation coefficient was determined. All models showed substantial correlation (ρ_c_ > 0.93) between the results of two independent observers regarding both, the absolute AAA volume and maximum aortic diameter. Reproducibility was lowest for volume analysis in the PPE model (ρ_c_ = 0.932). Supplementary Figures 1–4 illustrate correlations between the results of two observers in scatter plots (AAA volume and maximum aortic diameter in absolute measurements or percent aneurysm growth in relation to baseline) and provide both, the Spearman coefficient of correlation as well as Lin’s concordance correlation coefficient. Both parameters indicated higher variation for relative as compared to absolute values. Again, Lin’s concordance correlation coefficient was lowest for relative volume increase in the PPE model (ρ_c_ = 0.888, Supplementary Figure 4). Bland Altman plots (Supplementary Figures 5–8 and Table 1) depict the 95% limits of agreement for the four models; no trend toward increased observer deviation for larger aneurysm size was observed.

**TABLE 1:** Evaluation of reproducibility between two independent observers (inter-observer variation) in the AngII, ePPE ± BAPN and PPE model regarding 3D US measurements of AAA volume (mm^3^) and maximum aortic diameter (mm).

Model parameter	% Coefficient of variation: mean ± SD	95% Limits of agreement: lower, upper	Lin’s concordance correlation coefficient (95% CI)
**Inter-observer variation**			
AngII (*n* = 84*/77^#^)			
Volume (mm^3^)	4.46 ± 3.61	−3.04, 3.43	0.985 (0.977–0.990)
Diameter (mm)	4.49 ± 5.20	−0.33, 0.26	0.944 (0.915–0.964)
ePPE (*n* = 60*/58^#^)			
Volume (mm^3^)	1.65 ± 1.49	−0.52, 0.50	0.998 (0.997–0.999)
Diameter (mm)	3.30 ± 2.67	−0.12, 0.11	0.980 (0.968–0.988)
**ePPE + BAPN (*n* = 32)**			
Volume (mm^3^)	8.46 ± 5.40	−7.74, 3.79	0.952 (0.923–0.970)
Diameter (mm)	4.19 ± 3.38	−0.28, 0.21	0.979 (0.957–0.990)
**PPE (*n* = 37)**			
Volume (mm^3^)	5.42 ± 3.06	−0.85, 0.86	0.932 (0.873–0.964)
Diameter (mm)	3.42 ± 2.57	−0.11, 0.10	0.952 (0.909–0.975)
**Intra-observer variation**			
**ePPE + BAPN (*n* = 32)**			
Volume (mm^3^)	2.26 ± 2.60	−1.36, 1.62	0.998 (0.996–0.999)
Diameter (mm)	0.89 ± 0.67	−0.05, 0.05	0.992 (0.998–0.9996)

Intra-observer variation was additionally calculated for the ePPE + BAPN model. *Diverging analysis number for AAA volume vs. diameter**^#^**. 95% CI, 95% confidence intervals; SD, standard deviation.

### Differences Between Mouse Models in Time Course and Extent of Abdominal Aortic Aneurysm Development Are Reflected in Aortic Volume and Diameter Measurements

**FIGURE 3:**
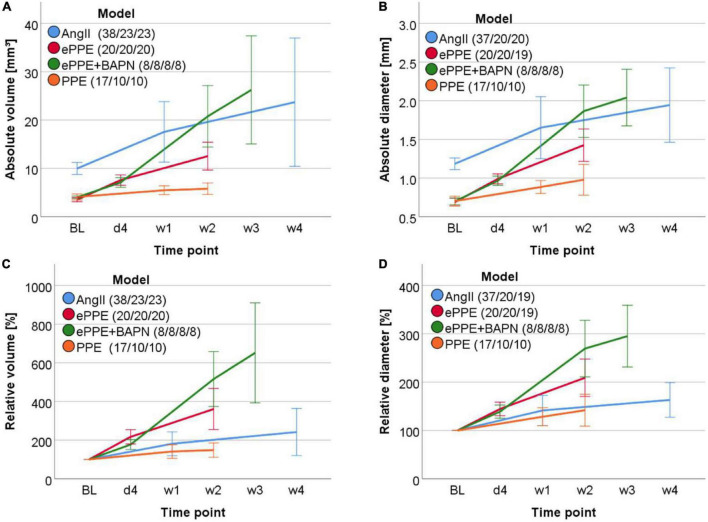
Time course of aorta expansion in four AAA mouse models. **(A)** Volume (mm^3^) over a 12 mm aortic stretch, **(B)** maximum aortic diameter (mm), **(C)** relative volume (%) in relation to baseline and **(D)** relative diameter (% of baseline) were measured by 3D US over time and are shown by line graphs. Time points included baseline (BL), day 4 after AAA induction (d4), week 1 (w1): days 6–8, w2: days 13–14, w3: day 21, w4: d27. Average measurements of two independent observers were applied. Variation between mice is illustrated by mean value ± standard deviation for each model and time point.

Initiation and progression of AAA in the four mouse models were detected by 3D ultrasound at distinct time points (Figure 1). The models differed substantially in their development of aneurysms with respect to absolute volume, absolute diameter, relative volume, and relative diameter (Figure 3 and Supplementary Figures 9–12). While all models performed in wildtype C57BL/6J animals showed comparable baseline values of aortic volume and diameter, the ApoE KO mice of the AngII model had higher starting values, in line with US measurement of the suprarenal as opposed to the infrarenal portion of the abdominal aorta (Figures 3A,B). In the AngII model (Supplementary Figure 9), aneurysms progressed from a mean baseline volume of 10.0 ± 1.2 to 17.6 ± 6.3 mm^3^ within 8 days to an end volume of 23.7 ± 13.3 mm^3^ after 4 weeks, which related to 181 ± 62 and 242 ± 122% growth of volume, respectively. The maximum aortic diameter increased from 1.2 ± 0.1 mm at baseline to 1.9 ± 0.5 mm at day 27, relating to a mean 163 ± 36% growth of diameter. The development of infrarenal aortic aneurysms in the ePPE model (Supplementary Figure 10) started from a mean of 3.5 ± 0.4 mm^3^ at baseline and progressed to 7.6 ± 1.1 mm^3^ by day 4 until 12.5 ± 2.9 mm^3^ after 2 weeks, relating to 217 ± 37 and 361 ± 106% mean growth of aortic volume, respectively. Comparably, the maximum diameter increased from 0.7 ± 0.05 to 1.4 ± 0.2 mm over 13 days, which corresponded to 209 ± 39% mean growth. In the ePPE + BAPN model (Supplementary Figure 11), absolute volume as well as absolute diameter at baseline were similar to the ePPE model (with mean values of 4.0 ± 0.3 mm^3^ and 0.7 ± 0.04 mm). However, the combined treatment triggered rapid and extensive aorta expansion as recorded on days 4, 14, and 21 to 7.1 ± 1.0, 20.8 ± 6.4, and 26.2 ± 11.2 mm^3^ volume and 1.0 ± 0.1, 1.9 ± 0.3, and 2.0 ± 0.4 mm diameter, respectively. This was also reflected in the highest relative growth of AAA volume and diameter, with a progression to 651 ± 259% of aortic volume and 295 ± 64% of maximum diameter on day 21. Lastly, the PPE model (Supplementary Figure 12) showed the lowest vessel expansion in comparison to the other three models, with the aortic volume starting at 4.1 ± 0.6 mm^3^ at baseline, increasing to 5.5 ± 0.9 mm^3^ after 1 week, and further to 5.8 ± 1.2 mm^3^ on day 13, relating to a mean 148 ± 37% growth of volume at the experimental endpoint. This is also reflected in maximum aortic diameter, starting at baseline with 0.7 ± 0.1 mm, and increasing to 0.9 ± 0.1 and 1.0 ± 0.2 mm on days 6 and 13, respectively, which translated to 142 ± 33% growth of aortic diameter over 2 weeks. Statistical LMEM analysis based on time as fixed effect and metric covariate revealed highly significant (*p* < 0.001) AAA growth in the AngII, ePPE, and ePPE + BAPN models for 3D volume as well as maximum diameter. In general, higher variation between experimental animals (random effect variance) was observed for relative as opposed to absolute measurements of volume or maximum diameter.

**FIGURE 4:**
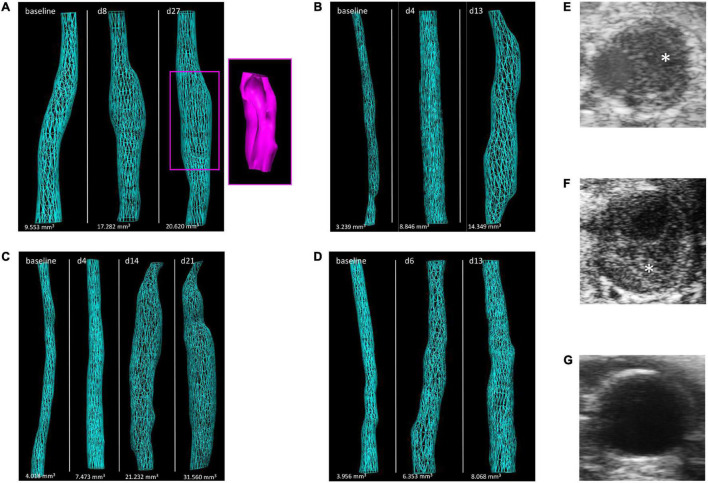
Illustration of morphological changes by 3D US over the time course of aneurysm development in the four AAA mouse models. Images of 3D reconstructed abdominal aorta regions are shown for representative mice of the **(A)** AngII model (including a 3D reconstructed thrombus in pink), **(B)** ePPE model, **(C)** ePPE + BAPN model, and **(D)** PPE model. Examples of aorta cross-sections in the AngII model are given for **(E,F)** AAAs with intramural thrombus (asterisk) or **(G)** without thrombus.

Importantly, the reconstructed 3D images of abdominal mouse aortas offered additional morphological information on the sequence of aneurysm formation. An example of early focal bulging and further aneurysm elongation is shown for the chronic AngII trigger (Figure 4A), while elastase based AAAs formed uniformly (over the entire treated aortic stretch) after the initial acute insult, followed by stronger expansion of distinct aorta regions in later development (Figures 4B–D). 3D reconstructions (Figure 4A) and cross-sectional images were also suited to detect intramural thrombi in dissected aortas of the AngII model (Figures 4E,F) which resulted in less discernable vessel walls when compared to AAAs without thrombus (Figure 4G).

### 3D Ultrasound Based Abdominal Aortic Aneurysm Monitoring Is More Effective in Detecting Early Aneurysm Growth Than by Conventional 2D B-Mode Analysis

Based on LMEM with time as fixed effect and categorical factor (comparing individual time points), a significant increase in both AAA volume and maximum aortic diameter was apparent for all four models at the investigated early and late time points of aneurysm development compared to baseline (Supplementary Figures 9–12). Significance was recorded for absolute as well as relative quantitation. Furthermore, with only one exception aneurysm expansion was also significant from the respective early to late time points of investigation. AAA volume (absolute or relative) did not increase significantly from days 6 to 13 in the PPE setting (Supplementary Figures 12A,C).

Since mouse count differed between the investigated models which affects recorded significance levels of aneurysm expansion, another tool was applied to quantify the separation between time points by standardized effect size (Cohen’s d) calculated as the difference between the means relative to their variability (Table 2). Based on this method distinct parameters such as volume and diameter can be more easily compared, and an effect size of 1.0 indicates that the means of the two time points (baseline and first AAA detection) are 1 standard deviation apart. Effect sizes differed between models, with ePPE ± BAPN scoring highest values between 3 and 4. With the exception of the PPE model, volume and diameter measurements derived from 3D US were mostly comparable in their effect size. Again, diameter was superior to volume assessment in the PPE group. Importantly, in this evaluation we also included maximum aortic diameter determined by conventional B-mode analysis. While 3D-derived parameters consistently reached the highest effect sizes, the difference to 2D B-mode analysis was most pronounced for the PPE model, where absolute diameter from 3D analysis reached an effect size of 1.61 as opposed to B-mode derived maximal diameter with 1.24.

**TABLE 2:** Standardized effect size for paired samples *t*-test based on Cohen’s calculation, comparing baseline to first time point of AAA measurement of absolute or relative volume and maximum aortic diameter in 3D US analysis as well as conventional 2D B-mode diameter assessment in all four mouse models.

Cohen’s d	AngII	ePPE	ePPE + BAPN	PPE
Absolute volume (mm^3^)	1.28	3.98	3.07	1.30
Relative volume (%)	1.32	3.17	3.03	1.16
Absolute diameter (mm)	1.30	3.70	3.27	1.61
Relative diameter (%)	1.33	3.19	2.85	1.52
Absolute 2D diameter (mm)	1.33	3.51	2.97	1.24
Relative 2D diameter (%)	1.31	3.13	2.66	1.25

### All Models Show a High Correlation Between 3D US Measurements of Abdominal Aortic Aneurysm Volume and Maximum Aortic Diameter Which Is in Good Agreement With *ex vivo* Aortic Diameter Assessment

**FIGURE 5:**
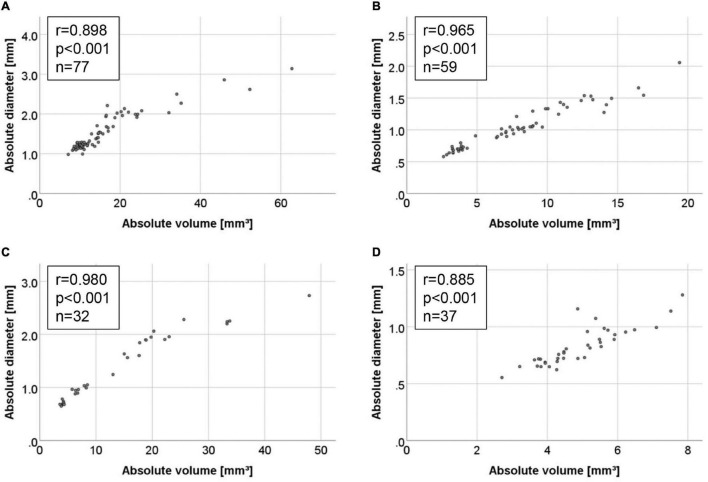
Correlations between absolute measurements of 3D volume and maximum aortic diameter in the four AAA mouse models. Correlations are illustrated by scattergram for the **(A)** AngII model, **(B)** ePPE model, **(C)** ePPE + BAPN model, and **(D)** PPE model. Spearman coefficient of correlation is given for all measured values, including baseline.

**FIGURE 6:**
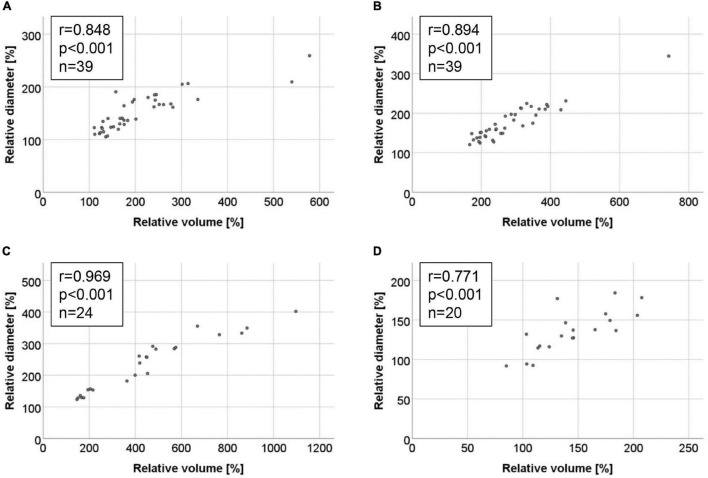
Correlations between relative increase in aneurysm volume and maximum aortic diameter in the four AAA mouse models. Correlations are illustrated by scattergram for the **(A)** AngII model, **(B)** ePPE model, **(C)** ePPE + BAPN model, and **(D)** PPE model. Baseline values were set to 100% and are hence excluded from the graph and Spearman coefficient of correlation.

Comparison of absolute volumes and diameters (Figure 5) as well as relative volumes and diameters (Figure 6) yielded high coefficients of correlation for all four AAA models. In general, the Spearman coefficient was highest for absolute measurements (*r* range from 0.885 to 0.980) with the ePPE + BAPN model reaching the highest score. In comparison, the association between relative increase in volume or diameter ranged between *r* = 0.771 and 0.969, again recording the highest Spearman coefficient of correlation for the ePPE + BAPN model.

**FIGURE 7:**
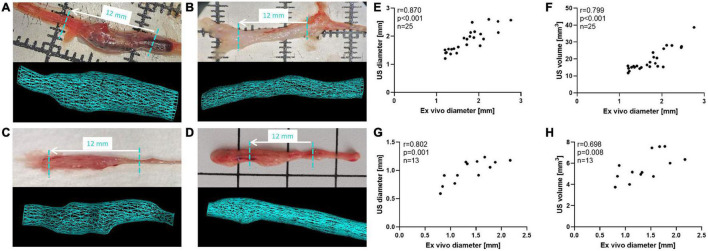
*Ex vivo* measurements of maximum aortic diameter compared to ultrasound derived AAA parameters. **(A–D)** Excised aortas are shown in top panels, while 3D reconstructed aorta volumes are illustrated in bottom panels for the **(A)** AngII model, **(B)** ePPE model, **(C)** ePPE + BAPN model, **(D)** PPE model. The length of US screened aorta regions (12 mm) is indicated in the respective images of excised aortas by a dashed turquoise line. **(E,G)** The correlation between maximum aortic diameter as determined by 3D US or measured *ex vivo* in the **(E)** AngII model and **(G)** PPE model were evaluated by Spearman coefficient of correlation. The comparison between *ex vivo* AAA diameter and 3D US volume is shown in **(F)** for the AngII model and **(H)** for the PPE model.

Finally, the correlations between maximum aortic diameter or volume derived from ultrasound and post mortem *ex vivo* measurement of aortic diameter were investigated in a subset of mice. In Figures 7A–D examples for ultrasound-derived 3D volumes are shown in comparison to excised aortas of the individual aneurysm models. Comparison between maximum aortic diameter inferred from ultrasound vs. *ex vivo* measurements yielded a Spearman coefficient of correlation of *r* = 0.870 in the AngII model (Figure 7E) and *r* = 0.802 in the PPE model (Figure 7G) as well as *r* = 0.786 in the ePPE model as we have previously reported (21). Correlations between *ex vivo* diameter and 3D US volume were somewhat weaker than for US derived diameter in the AngII model (*r* = 0.799; Figure 7F) and substantially lower in the PPE model (*r* = 0.698; Figure 7H). When we compared AAA volume as measured by 3D US to aneurysm volume determined by serial diameter data of the excised aortas, the coefficients of correlation ranged at *r* = 0.680 for the AngII model and *r* = 0.607 for the PPE model (Supplementary Figure 13).

## Discussion

Goldberg et al. were the first to compare 3D US with 3D histological AAA reconstruction (based on *ex vivo* stained aortic sections) in a specific allograft-based mouse model (22). While the study was exploratory and limited to end-point analysis of 6 mice, the authors convincingly demonstrated the reliability of 3D US to reflect AAA volume (outer wall and inner lumen) and morphological features like aortic plaques as confirmed by histological analysis. They advised 3D US to be evaluated in other AAA models and in longitudinal measurement of disease progression (22). Waduud et al. evolved the method of 3D US based AAA analysis by applying the fast and semi-automated acquisition of 157 frames over a 12 mm stretch with subsequent detailed manual contouring for 3D aneurysm reconstruction (20). While Waduud et al. provided proof-of-principle for the sensitive detection of AAA formation by 3D US in the ePPE model, our study allowed us to elucidate the limitations of 3D volume analysis by comparing the time course of aneurysm formation in four commonly applied AAA mouse models: Although 3D US generally proved more effective than conventional 2D B-mode in detecting early AAA formation, the 3D based volume analysis was found inferior to maximum diameter assessment in a surgical AAA model where the region of local injury is crucially determined by the individual anatomical features (PPE model).

Thus, while the mouse models differed substantially in the time course and extent of aneurysm formation, the analysis via 3D ultrasound yielded highly reproducible and interobserver-robust growth measurements of aortic volume and maximum diameter as reliable outcome parameters for *in vivo* studies. As demonstrated by the CV (Table 1), volume as well as diameter measurements in all four models showed little deviation between two independent observers. The highest variability of 8.46 ± 5.40% was recorded for the volume assessment in the ePPE + BAPN model, which is likely due to the substantially larger aneurysm size and occurrence of thrombi in this setting as opposed to ePPE without BAPN supplementation which showed the lowest interobserver variation and highest concordance coefficient of correlation (ρ_c_ = 0.998). In line, the Spearman coefficient of correlation between two independent assessments generally ranged ≥ 0.9 for all models and was consistently higher for absolute volume or diameter measurements compared to relative increase (Supplementary Figures 1–8).

The distinct growth patterns of AAA models depicted in Figure 3 have previously been reported by other groups and are likely due to the different triggers and pathogenic mechanisms of the respective models (13, 14, 16, 17). We have found that in three of the four investigated models (AngII, ePPE ± BAPN), in the initiation as well as in the AAA progression phase, there was a significant increase in absolute and relative volume and diameter over the commonly applied experimental time frames (Supplementary Figures 9–12). However, regarding the volumetric measurements of the PPE model this did not hold true, i.e., after the initial vessel expansion over 6 days the aortic volume did not rise significantly during the second experimental week. Also, the progression of maximum aortic diameter was minimal, reaching 128% on day 6 and 142% on day 13. This observation is in contrast to the first published landmark paper of aortic perfusion in mice, where immediately after elastase instillation the maximum aortic diameter increased to 174%, remained at this size until day 7 but reached 234% of baseline after 2 weeks (13). Of note, the group used a different mouse strain (129/SvJ mice), higher sample size (*n* = 33) and measured the aortic diameter *in situ* with a calibrated ocular grid, which might account for the AAA growth pattern distinct from our PPE results.

Furthermore, it should be noted that doubling in maximum aortic diameter has previously been applied as a cut-off in AAA mouse studies but does not match the human aneurysm definition (50% increase). While this threshold was frequently unmet in the PPE and AngII models, the presented 3D ultrasound analysis offers the tools to detect significant aorta expansion with high sensitivity and little measurement error. Thus, AAA growth may be reliably determined in mice with less than doubled diameter which may help to reduce animal numbers in experiments.

With respect to the newly introduced method of 3D ultrasound all mouse models showed good agreement between measurements of aortic volume and diameter (Figures 5, 6), and the 3D US inferred parameters corresponded well with *ex vivo* measurements of excised aneurysms (Figure 7). Again, absolute rather than relative AAA growth yielded a better correlation between 3D US derived volume and diameter measurements. In comparison to the other mouse models, PPE resulted in the lowest rate of aortic expansion with better interobserver consistency for AAA diameter than volume measurements, and higher effect size for early aneurysm detection by diameter than volume analysis. For the PPE model, 3D US derived diameter rather than volume correlated with *ex vivo* AAA analysis. We propose that the technical nature of the model might account for this distinctiveness. The anatomical conditions greatly determine the portion of the infrarenal mouse aorta which can be dissected and ligated for *in vivo* perfusion with elastase. We observed a high variation in the anatomy of murine abdominal aortas, regarding amount and localization of side as well as lumbar branches. Since the placement of ligatures defines the length of the aorta injury, this introduces a bias regarding the volume of AAA formation while maximum vessel enlargement in terms of aortic diameter is more consistent.

Thus, in addition to the low interobserver variability and easy application of 3D US across different laboratories, the strength of this technique certainly lies within the high sensitivity in monitoring aorta expansion at the early time points. Of note, the relative increase (in percent of baseline) was found to be substantially higher for volume than diameter in all investigated models: 181% vs. 141% (AngII), 217% vs. 145% (ePPE), 177% vs. 139% (BAPN), and 141% vs. 128% (PPE). Furthermore, when compared to conventional 2D B-mode analysis, the highest (standardized) effect size was generally recorded for the 3D US derived parameters to detect significant AAA growth at an early time point (Table 2) which may facilitate animal stratification into treatment groups and improve the assessment of eventual regression upon drug therapy. Moreover, 3D volume reconstruction does not only assist in identifying the site of maximum aortic diameter but yields more detailed information about the underlying morphology of the aneurysm. In line with the distinct pathological triggers, the mouse models exhibited major differences in the shaping aneurysms, and monitoring the entire aneurysm rather than maximum diameter might offer more information on potential drug effects. When comparing 3D preclinical ultrasound to the clinical situation where manual 2D readout is generally applied, the experimental results encourage the implementation of a software-assisted, semi-automated output of AAA volume and maximum aortic diameter for the coherent monitoring of aneurysm growth by ultrasound as is increasingly supported by studies (23–25).

### Limitations

As the study was retrospective in design, the number of available data sets differed between the investigated mouse models. Thus, the variation in sample size limits comparability between models but does not impair the assessment of volume vs. diameter within each model. Of note, effect size (Cohen’s d) was comparable for the ePPE (*n* = 20) and ePPE + BAPN (*n* = 8) models despite the substantial difference in animal numbers.

While we found 3D based approaches to be highly reproducibly and particularly effective in detecting early formed aneurysms, the 3D US image recording and aorta reconstruction does require considerably more time than conventional 2D B-mode analysis. Also, expenses for aorta scans by high frequency ultrasound systems have to be considered (5–7 min per mouse).

## Data Availability Statement

The raw data supporting the conclusions of this article will be made available by the authors, without undue reservation.

## Ethics Statement

The animal study was reviewed and approved by the Ethics Committee of the Medical University of Vienna for animal experiments and by the Austrian Ministry of Science.

## Author Contributions

NI, SB, WE, CN, MB, and CB: conceptualization (ideas; formulation of research goals and aims). NI, SB, JK, VK, and AB: investigation (conducting experiments). NI, SB, JK, VK, HH, GK, and AS-T: sample analysis (collecting and analyzing data). NI, SB, and CB: formal analysis (application of statistical techniques to analyze study data). MB and CB: funding acquisition. NI, SB, JK, AB, and MB: methodology (development or design of methods). SB, NI, and CB: writing (original draft preparation). All authors contributed to the writing (review and editing).

## Conflict of Interest

The authors declare that the research was conducted in the absence of any commercial or financial relationships that could be construed as a potential conflict of interest.

## Publisher’s Note

All claims expressed in this article are solely those of the authors and do not necessarily represent those of their affiliated organizations, or those of the publisher, the editors and the reviewers. Any product that may be evaluated in this article, or claim that may be made by its manufacturer, is not guaranteed or endorsed by the publisher.
