# Supplementary material for: 3D Ultrasound Measurements Are Highly Sensitive to Monitor Formation and Progression of Abdominal Aortic Aneurysms in Mouse Models
Source: Front Cardiovasc Med. 2022 Jul 12;9:944180. doi: 10.3389/fcvm.2022.944180 (PMC9314770; doi:10.3389/fcvm.2022.944180)
Supplement: Supplementary file 1 [file Data_Sheet_1.pdf]

frontiers

Supplementary Material

3D Ultrasound Measurements Are Highly Sensitive to Monitor Formation and Progression of Abdominal Aortic Aneurysms in Mouse Models

Nahla Ibrahim[1], Sonja Bleichert[1], Johannes Klopf[1], Gabriel Kurzreiter[1], Viktoria Knöbl[1], Hubert Hayden[1], Albert Busch[2], Alexander Stiglbauer-Tscholakoff[3], Wolf Eilenberg[1], Christoph Neumayer[1], Marc A. Bailey[4,5], and Christine Brostjan[1]*

[1]Division of Vascular Surgery, Department of General Surgery, Medical University of Vienna and Vienna General Hospital, Vienna, Austria

[2]Department for Visceral, Thoracic and Vascular Surgery, Technical University of Dresden and University Hospital Carl-Gustav Carus, Dresden, Germany

[3]Division of Cardiovascular and Interventional Radiology, Division of Molecular and Gender Imaging, Department of Biomedical Imaging and Image Guided Therapy, Medical University of Vienna and Vienna General Hospital, Vienna, Austria

[4]Leeds Institute for Cardiovascular and Metabolic Medicine, School of Medicine, University of Leeds, Leeds, United Kingdom

[5]Leeds Vascular Institute, Leeds General Infirmary, Great George Street, Leeds, United Kingdom

*** Correspondence:**
Prof. Christine Brostjan, PhD
christine.brostjan@meduniwien.ac.at

SUPPLEMENTARY FIGURE LEGENDS

Suppl. Figure 1: Interobserver correlations in the AngII model regarding four aortic parameters in 3D ultrasound analysis. A ǀ Absolute volume [mm^3]. B ǀ Absolute diameter [mm]. C ǀ Relative volume [% of baseline]. D ǀ Relative diameter [% of baseline]. Spearman coefficient of correlation r and Lin's concordance correlation coefficient ρ with 95% confidence intervals (95% CI) are given.

Suppl. Figure 2: Interobserver correlations in the ePPE model regarding four aortic parameters in 3D ultrasound analysis. A ǀ Absolute volume [mm^3]. B ǀ Absolute diameter [mm]. C ǀ Relative volume [% of baseline]. D ǀ Relative diameter [% of baseline]. Spearman coefficient of correlation r and Lin's concordance correlation coefficient ρ with 95% confidence intervals (95% CI) are given.

Suppl. Figure 3: Interobserver correlations in the ePPE+BAPN model regarding four aortic parameters in 3D ultrasound analysis. A ǀ Absolute volume [mm^3]. B ǀ Absolute diameter [mm]. C ǀ Relative volume [% of baseline]. D ǀ Relative diameter [% of baseline]. Spearman coefficient of correlation r and Lin's concordance correlation coefficient ρ with 95% confidence intervals (95% CI) are given.

Suppl. Figure 4: Interobserver correlations in the PPE model regarding four aortic parameters in 3D ultrasound analysis. A ǀ Absolute volume [mm^3]. B ǀ Absolute diameter [mm]. C ǀ Relative volume [% of baseline]. D ǀ Relative diameter [% of baseline]. Spearman coefficient of correlation r and Lin's concordance correlation coefficient ρ with 95% confidence intervals (95% CI) are given.

Suppl. Figure 5: Interobserver differences depicted by Bland Altman plots for the AngII model regarding four aortic parameters in 3D ultrasound analysis. A ǀ Absolute volume [mm^3]. B ǀ Absolute diameter [mm]. C ǀ Relative volume [% of baseline]. D ǀ Relative diameter [% of baseline].

Suppl. Figure 6: Interobserver differences depicted by Bland Altman plots for the ePPE model regarding four aortic parameters in 3D ultrasound analysis. A ǀ Absolute volume [mm^3]. B ǀ Absolute diameter [mm]. C ǀ Relative volume [% of baseline]. D ǀ Relative diameter [% of baseline].

Suppl. Figure 7: Interobserver differences depicted by Bland Altman plots for the ePPE+BAPN model regarding four aortic parameters in 3D ultrasound analysis. A ǀ Absolute volume [mm^3]. B ǀ Absolute diameter [mm]. C ǀ Relative volume [% of baseline]. D ǀ Relative diameter [% of baseline].

Suppl. Figure 8: Interobserver differences depicted by Bland Altman plots for the PPE model regarding four aortic parameters in 3D ultrasound analysis. A ǀ Absolute volume [mm^3]. B ǀ Absolute diameter [mm]. C ǀ Relative volume [% of baseline]. D ǀ Relative diameter [% of baseline].

Suppl. Figure 9: Development of suprarenal AAAs over time in the AngII model. The development of a suprarenal AAA over time was investigated in the AngII model as measured by A ǀ absolute volume [mm^3], B ǀ absolute diameter [mm], C ǀ relative volume [% of baseline] and D ǀ relative diameter [% of baseline]. Displayed are mean ± standard deviation and single data points (n=38/23/23 mice). LMEM analysis with time as metric covariate is summarized by beta values (fixed effect estimates), 95% confidence limits (95% CI) and random effect variance. P-values indicated in graphs by * $p<0.05$, ** $p<0.01$, *** $p<0.001$ refer to LMEM analysis with time as categorical factor for comparison of individual time points.

Suppl. Figure 10: Development of infrarenal AAAs over time in the ePPE model. The development of an infrarenal AAA over time was investigated in the ePPE model (n=20 mice) as measured by A ǀ absolute volume [mm^3], B ǀ absolute diameter [mm], C ǀ relative volume [% of baseline] and D ǀ relative diameter [% of baseline]. LMEM analysis with time as metric covariate is summarized by beta values (fixed effect estimates), 95% confidence limits (95% CI) and random effect variance. P-values indicated in graphs by * $p<0.05$, ** $p<0.01$, *** $p<0.001$ refer to LMEM analysis with time as categorical factor for comparison of individual time points.

Suppl. Figure 11: Development of infrarenal AAAs over time in the ePPE+BAPN model. The development of an infrarenal AAA over time was investigated in the ePPE+BAPN model (n=8 mice) as measured by A ǀ absolute volume [mm^3], B ǀ absolute diameter [mm], C ǀ relative volume [% of baseline] and D ǀ relative diameter [% of baseline]. LMEM analysis with time as metric covariate is summarized by beta values (fixed effect estimates), 95% confidence limits (95% CI) and random effect variance. P-values indicated in graphs by * $p<0.05$, ** $p<0.01$, *** $p<0.001$ refer to LMEM analysis with time as categorical factor for comparison of individual time points.

Suppl. Figure 12: Development of infrarenal AAAs over time in the PPE model. The development of an infrarenal AAA over time was investigated in the PPE model as measured by A ǀ absolute volume [mm^3], B ǀ absolute diameter [mm], C ǀ relative volume [% of baseline] and D ǀ relative diameter [% of baseline]. Displayed are mean ± standard deviation and single data points (n=17/10/10 mice). LMEM analysis with time as metric covariate is summarized by beta values (fixed effect estimates), 95% confidence limits (95% CI) and random effect variance. P-values indicated in graphs by * $p<0.05$, ** $p<0.01$, *** $p<0.001$ refer to LMEM analysis with time as categorical factor for comparison of individual time points (ns, not significant).

Suppl. Figure 13: *Ex vivo* measurements of AAA volume compared to ultrasound derived aneurysm volume. The correlation between aneurysm volume as measured by 3D US or determined *ex vivo* by serial diameter measurements in the A ǀ AngII model (n=25) and B ǀ PPE model (n=7) were evaluated by Spearman coefficient of correlation.

Suppl. Fig. 1

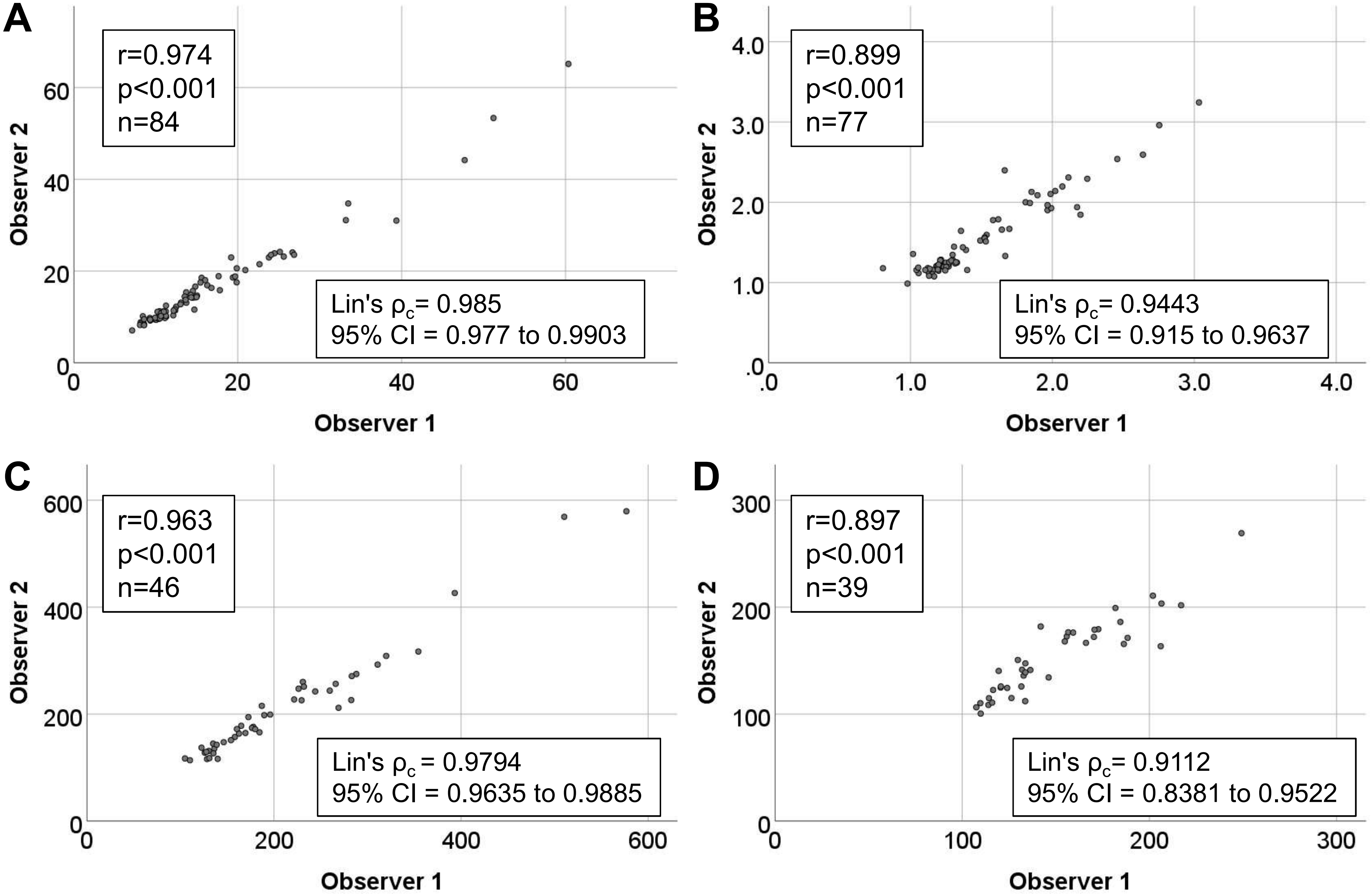
A
r=0.974
p<0.001
n=84
Lin's ρc= 0.985
95% CI = 0.977 to 0.9903
Observer 2
Observer 1
B
r=0.899
p<0.001
n=77
Lin's ρc= 0.9443
95% CI = 0.915 to 0.9637
Observer 2
Observer 1
C
r=0.963
p<0.001
n=46
Lin's ρc = 0.9794
95% CI = 0.9635 to 0.9885
Observer 2
Observer 1
D
r=0.897
p<0.001
n=39
Lin's ρc= 0.9112
95% CI = 0.8381 to 0.9522
Observer 2
Observer 1

Suppl. Fig. 2

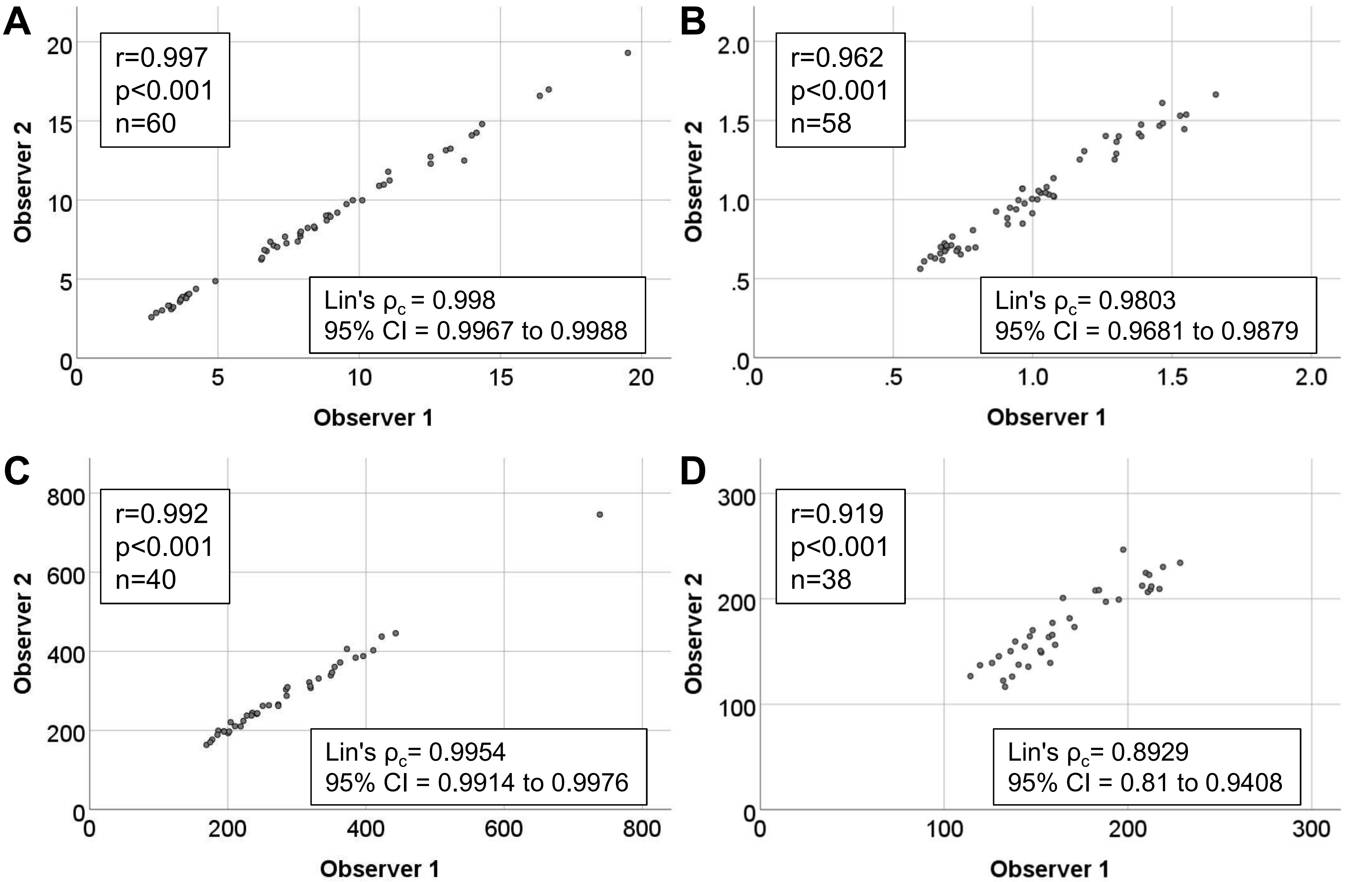
A
r=0.997
p<0.001
n=60
Lin's ρ_c = 0.998
95% CI = 0.9967 to 0.9988
Observer 2
Observer 1
B
r=0.962
p<0.001
n=58
Lin's ρ_c= 0.9803
95% CI = 0.9681 to 0.9879
Observer 2
Observer 1
C
r=0.992
p<0.001
n=40
Lin's ρ_c= 0.9954
95% CI = 0.9914 to 0.9976
Observer 2
Observer 1
D
r=0.919
p<0.001
n=38
Lin's ρ_c= 0.8929
95% CI = 0.81 to 0.9408
Observer 2
Observer 1

Suppl. Fig. 3

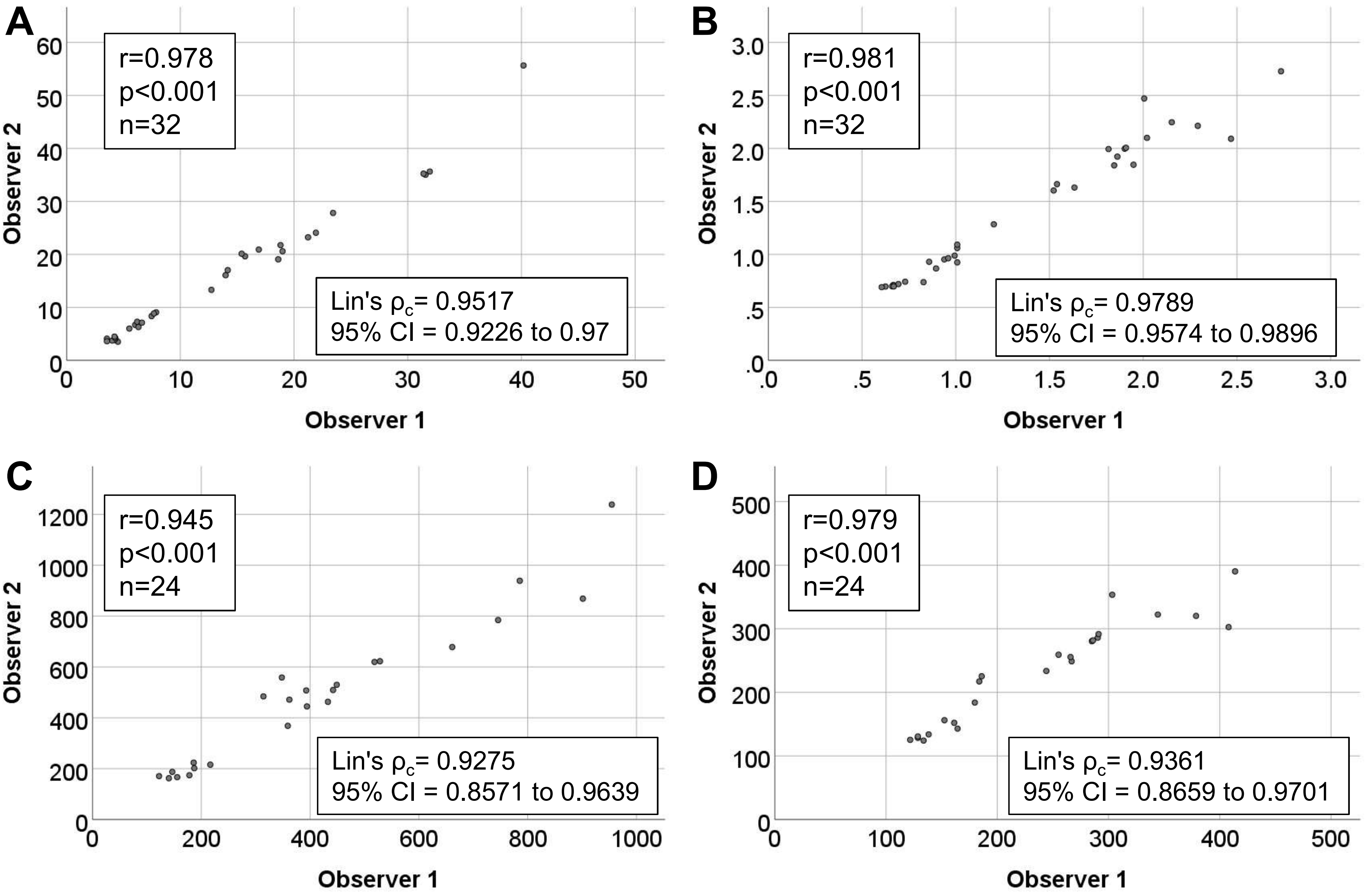
A
r=0.978
p<0.001
n=32
Lin's ρc= 0.9517
95% CI = 0.9226 to 0.97
Observer 1
Observer 2
B
r=0.981
p<0.001
n=32
Lin's ρc= 0.9789
95% CI = 0.9574 to 0.9896
Observer 1
Observer 2
C
r=0.945
p<0.001
n=24
Lin's ρc= 0.9275
95% CI = 0.8571 to 0.9639
Observer 1
Observer 2
D
r=0.979
p<0.001
n=24
Lin's ρc= 0.9361
95% CI = 0.8659 to 0.9701
Observer 1
Observer 2

Suppl. Fig. 4

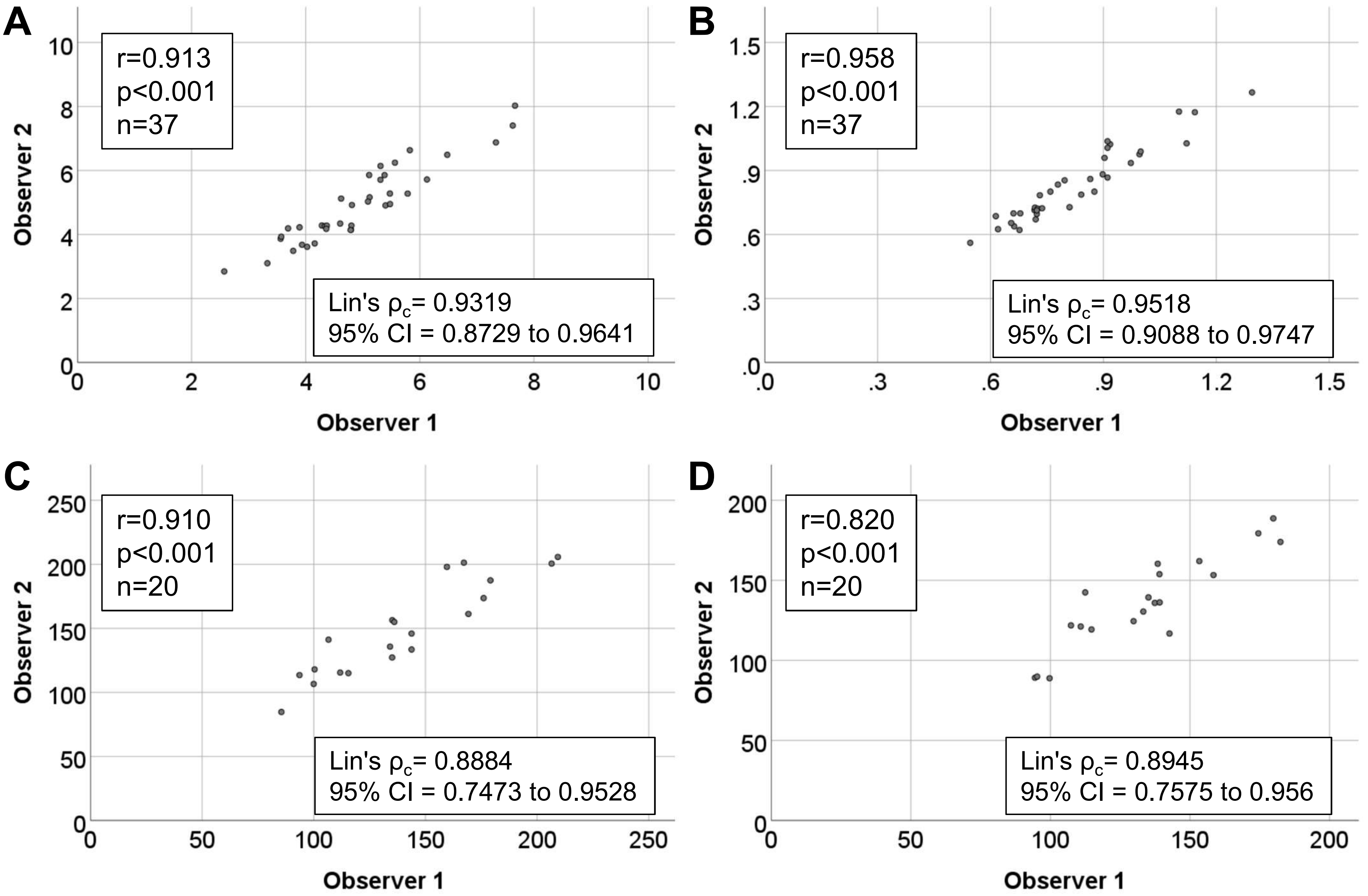
A
r=0.913
p<0.001
n=37
Lin's ρ_c= 0.9319
95% CI = 0.8729 to 0.9641
Observer 2
Observer 1
B
r=0.958
p<0.001
n=37
Lin's ρ_c= 0.9518
95% CI = 0.9088 to 0.9747
C
r=0.910
p<0.001
n=20
Lin's ρ_c= 0.8884
95% CI = 0.7473 to 0.9528
D
r=0.820
p<0.001
n=20
Lin's ρ_c= 0.8945
95% CI = 0.7575 to 0.956

Suppl. Fig. 5

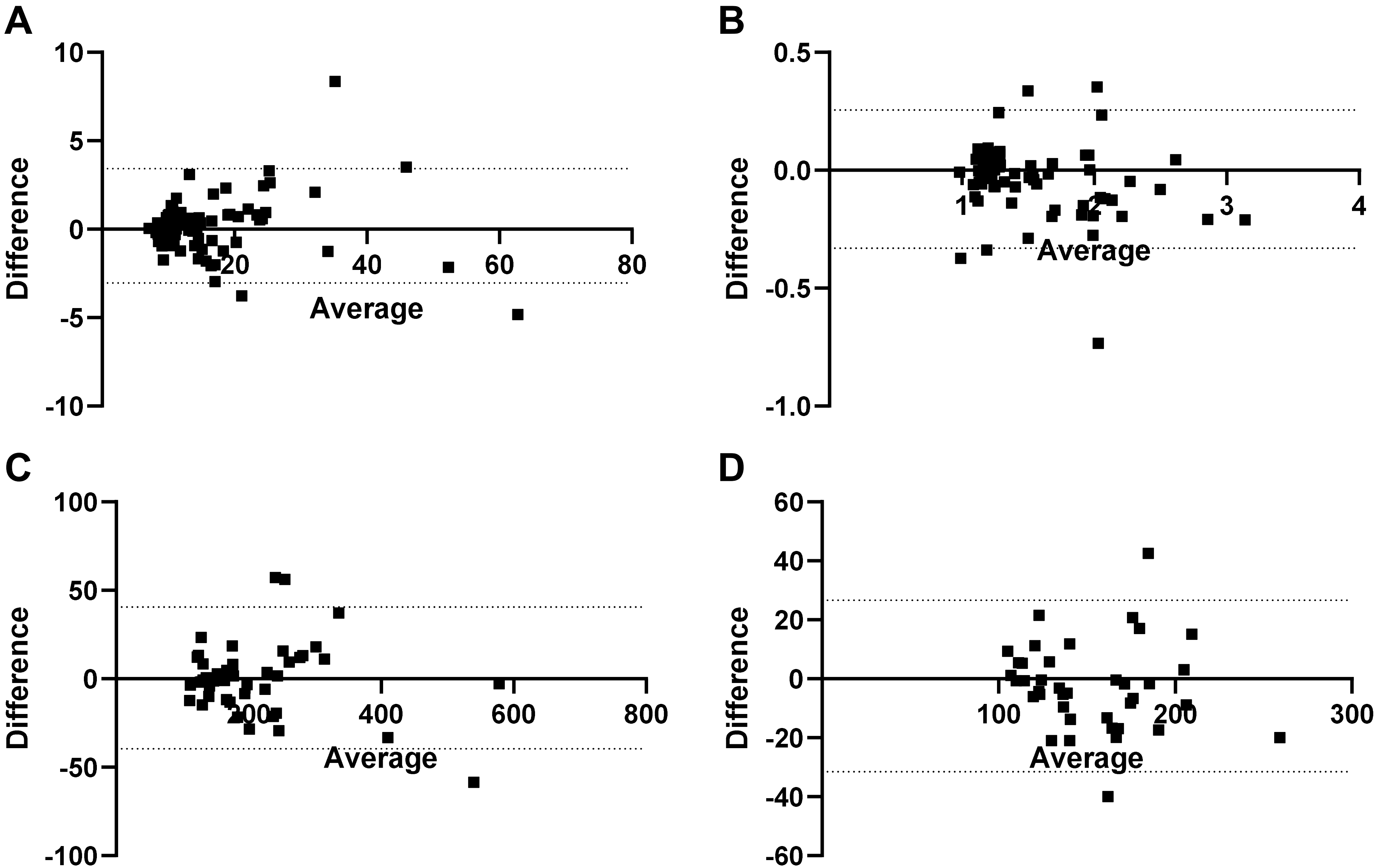
A
B
C
D
Difference
Average
10
5
0
-5
-10
20
40
60
80
0.5
0.0
-0.5
-1.0
1
2
3
4
100
50
-50
-100
200
400
600
800
60
40
-20
-40
-60
300

Suppl. Fig. 6

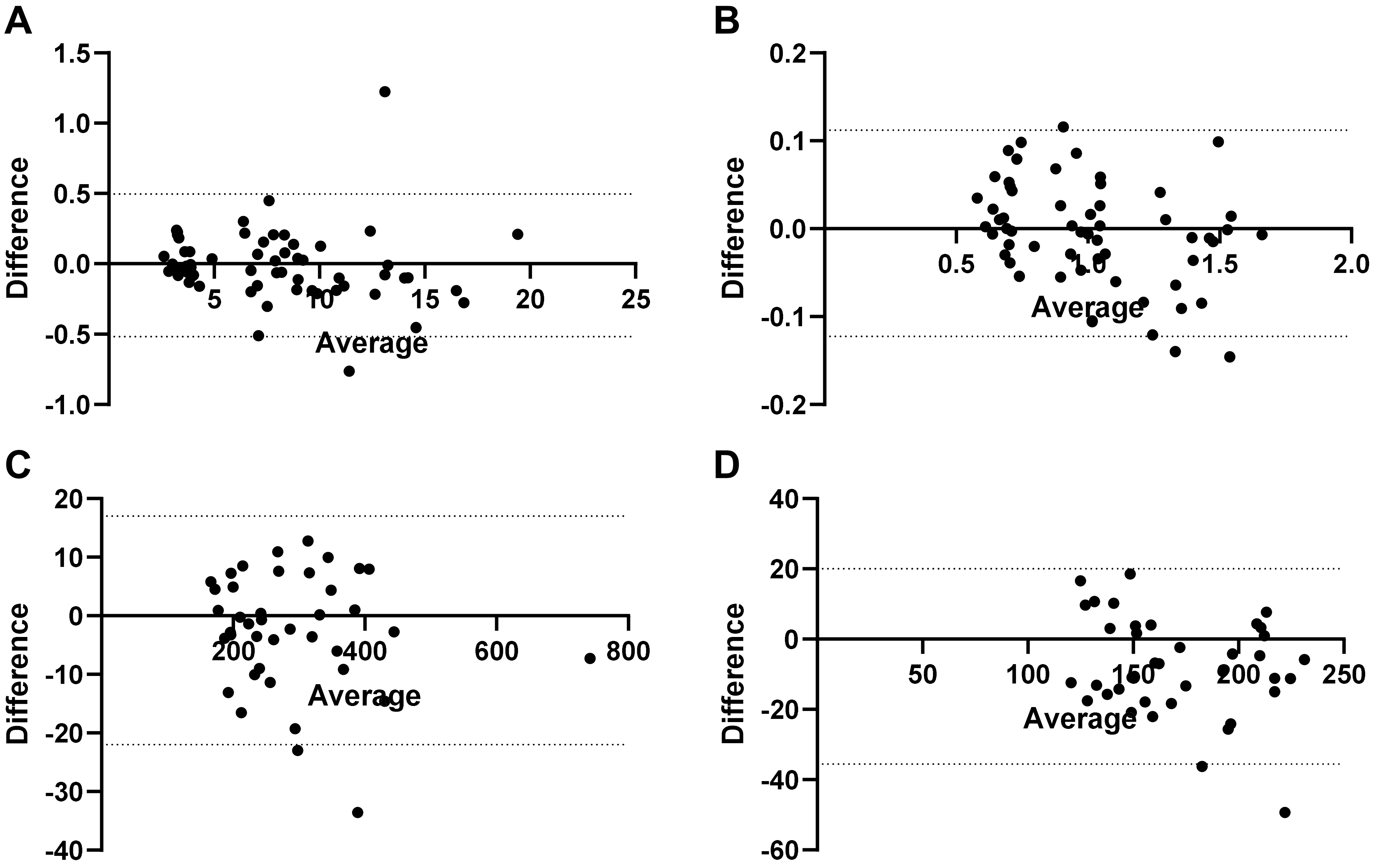
A
1.5
1.0
0.5
0.0
-0.5
-1.0
Difference
5
10
15
20
25
Average
B
0.2
0.1
0.0
-0.1
-0.2
Difference
0.5
1.0
1.5
2.0
Average
C
20
10
0
-10
-20
-30
-40
Difference
200
400
600
800
Average
D
40
20
0
-20
-40
-60
Difference
50
100
150
200
250
Average

Suppl. Fig. 7

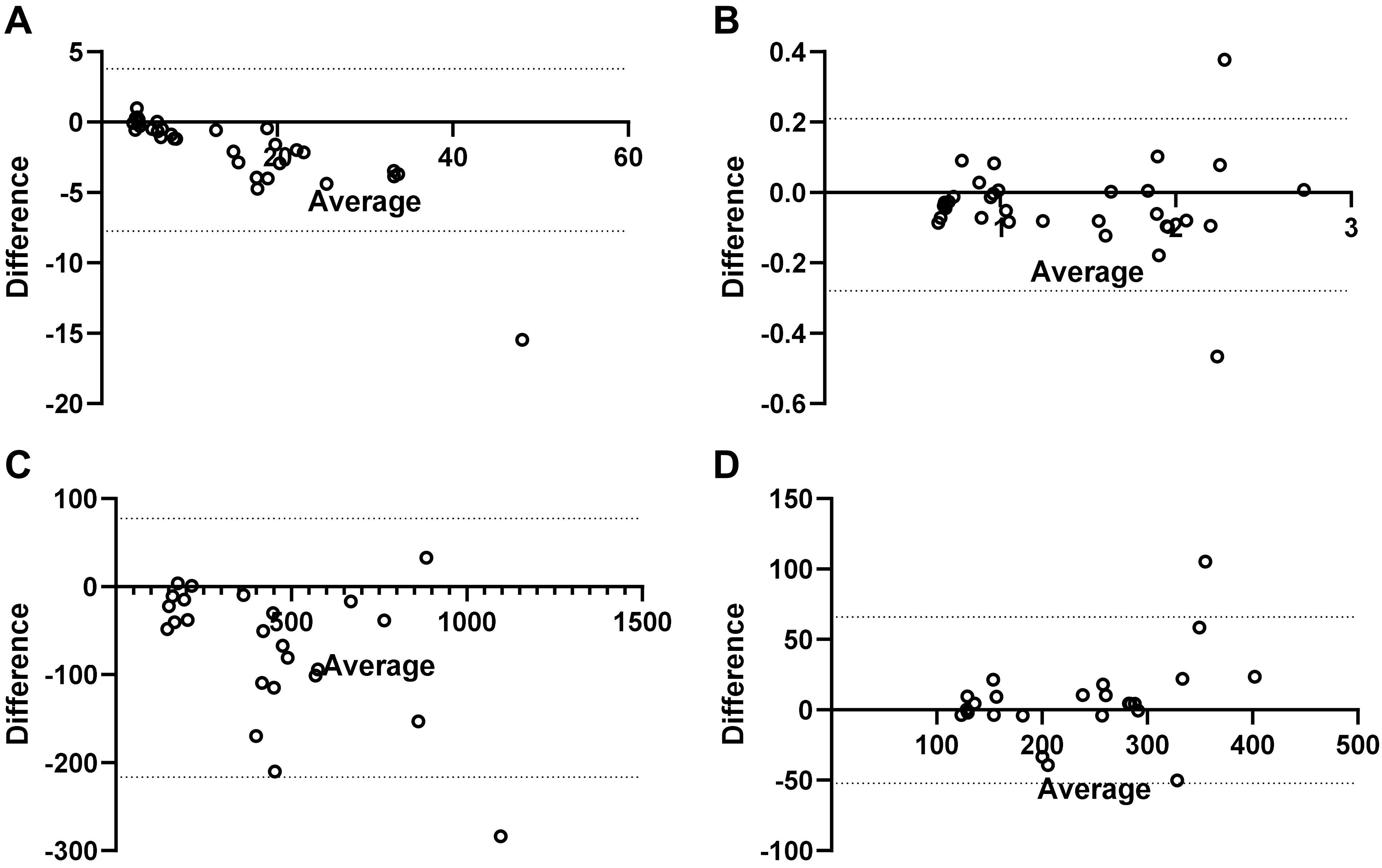
A
Difference
Average
5
0
-5
-10
-15
-20
20
40
60
B
Difference
Average
0.4
0.2
0.0
-0.2
-0.4
-0.6
1
2
3
C
Difference
Average
100
0
-100
-200
-300
500
1000
1500
D
Difference
Average
150
100
50
0
-50
-100
100
200
300
400
500

Suppl. Fig. 8

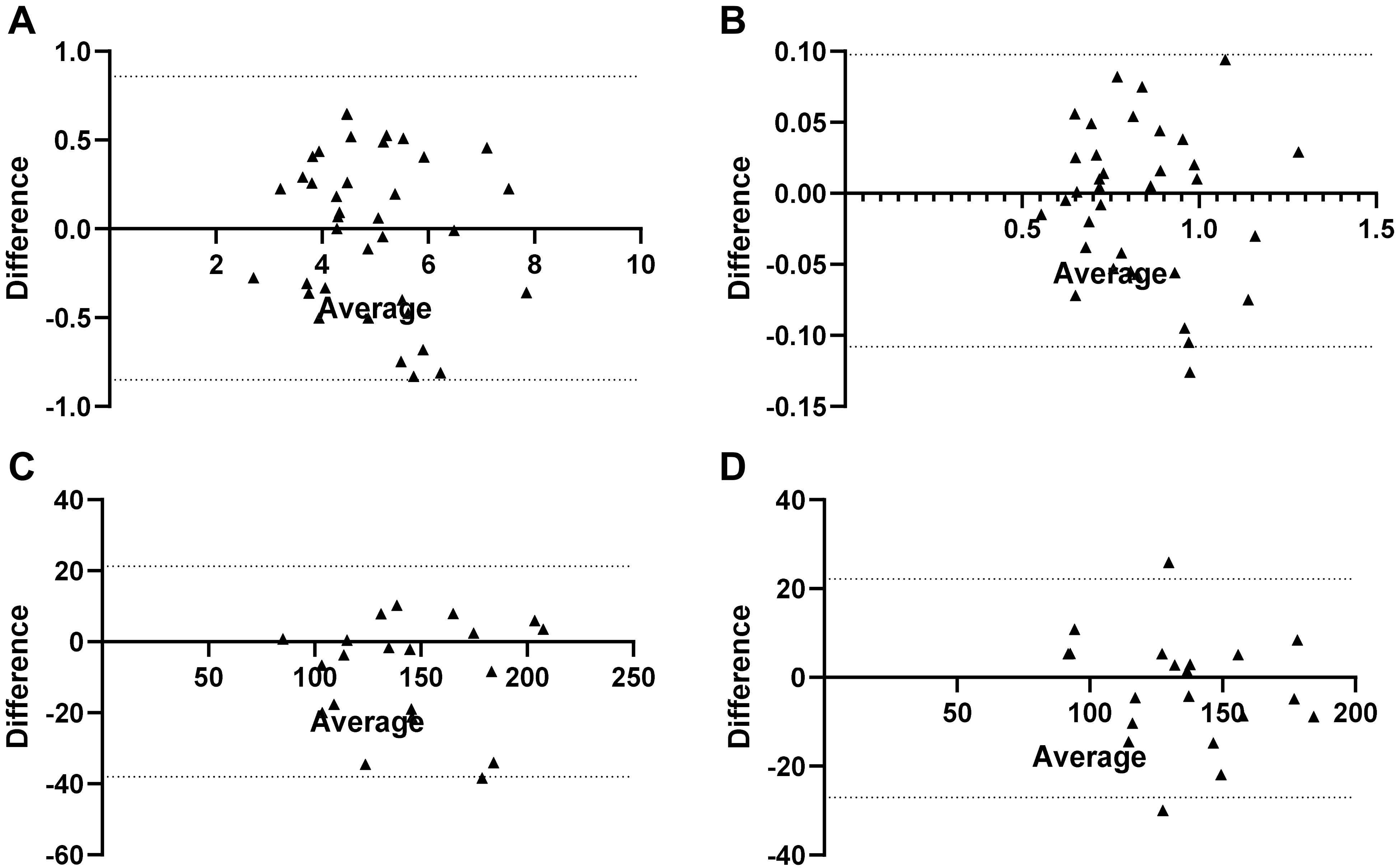
A
B
C
D
Difference
Average
1.0
0.5
0.0
-0.5
-1.0
2
4
6
8
10
0.10
0.05
0.00
-0.05
-0.10
-0.15
0.5
1.0
1.5
40
20
0
-20
-40
-60
50
100
150
200
250

Suppl. Fig. 9

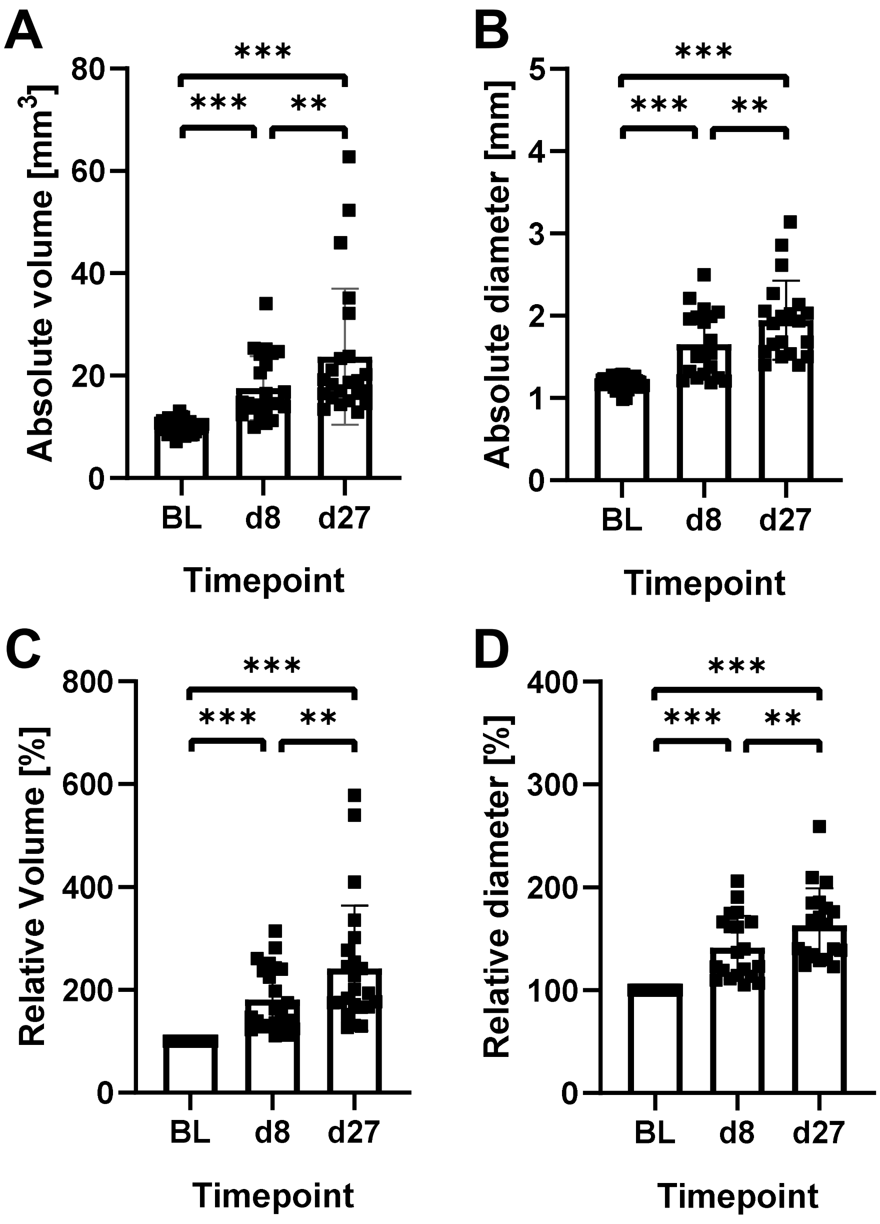

LMEM	P-value	Beta	Lower 95% CI	Upper 95% CI	Random effect
A	< 0.001	0.492	0.359	0.625	15.416
B	< 0.001	0.027	0.021	0.033	0.027
C	< 0.001	5.062	3.787	6.337	1143.39
D	< 0.001	2.266	1.763	2.770	105.76

Suppl. Fig. 10

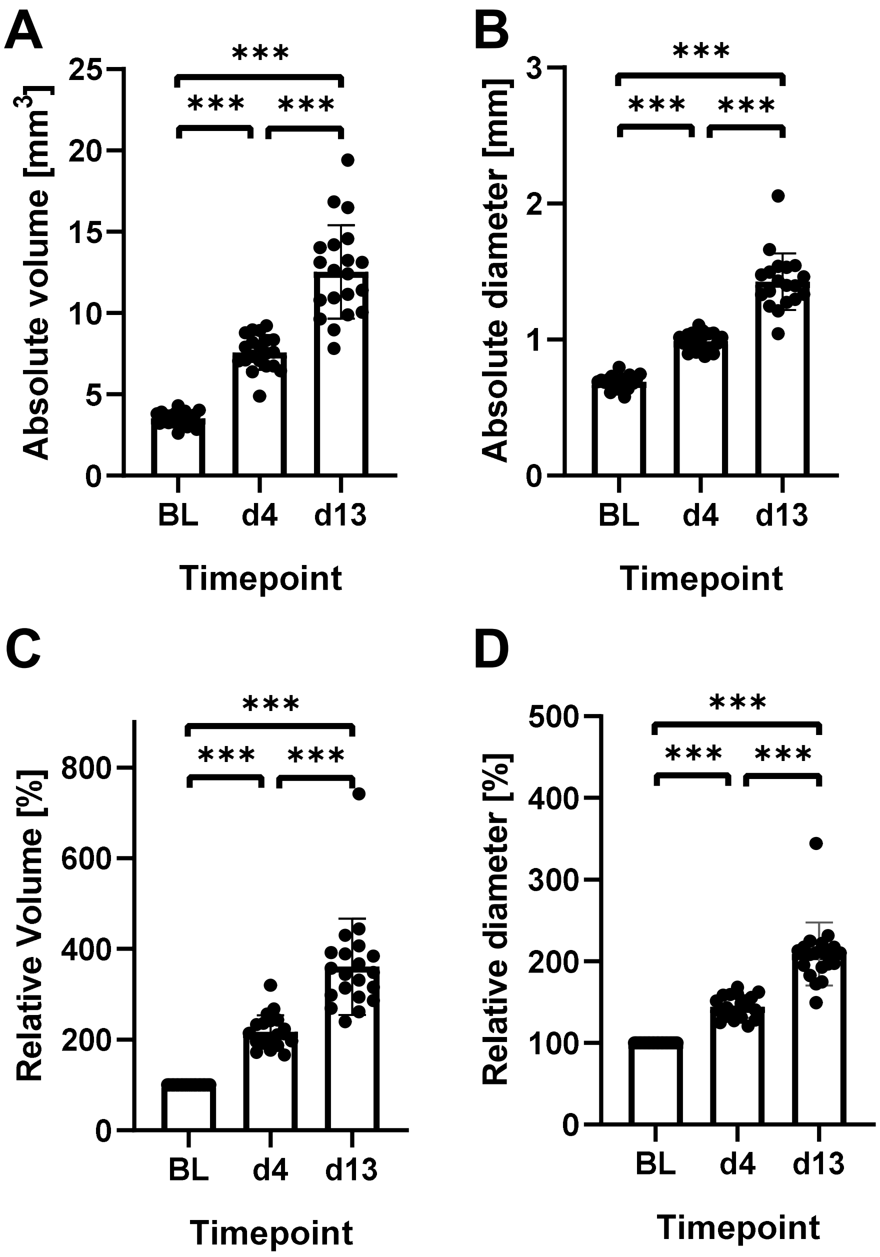

LMEM	P-value	Beta	Lower 95% CI	Upper 95% CI	Random effect
A	< 0.001	0.669	0.588	0.750	0.662
B	< 0.001	0.055	0.049	0.062	0.000
C	< 0.001	19.369	16.527	22.212	949.06
D	< 0.001	8.184	7.128	9.240	100.02

Suppl. Fig. 11

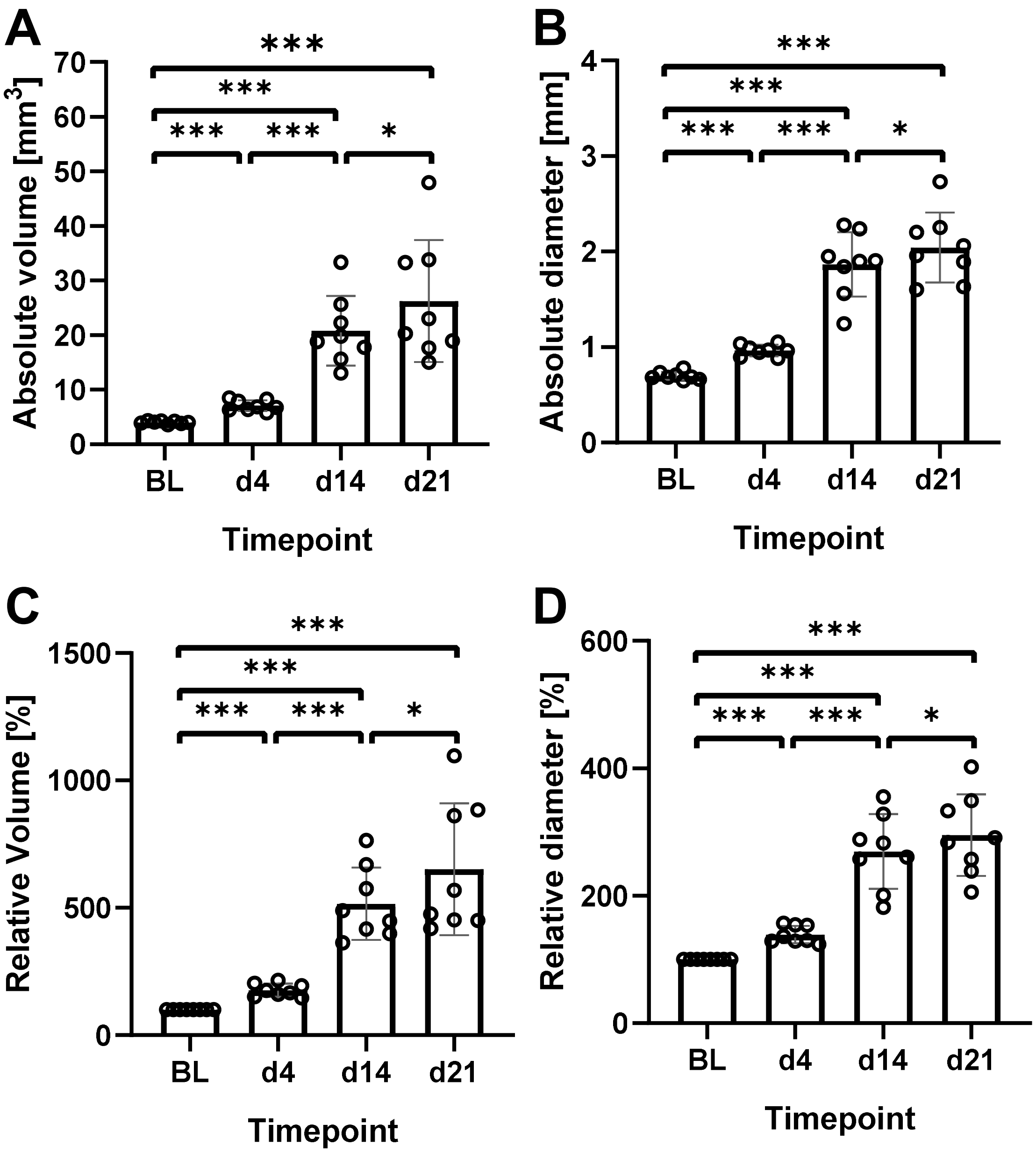

LMEM	P-value	Beta	Lower 95% CI	Upper 95% CI	Random effect
A	< 0.001	1.113	0.886	1.339	14.745
B	< 0.001	0.068	0.057	0.078	0.016
C	< 0.001	27.596	22.320	32.872	7435.82
D	< 0.001	9.872	8.216	11.527	723.53

Suppl. Fig. 12

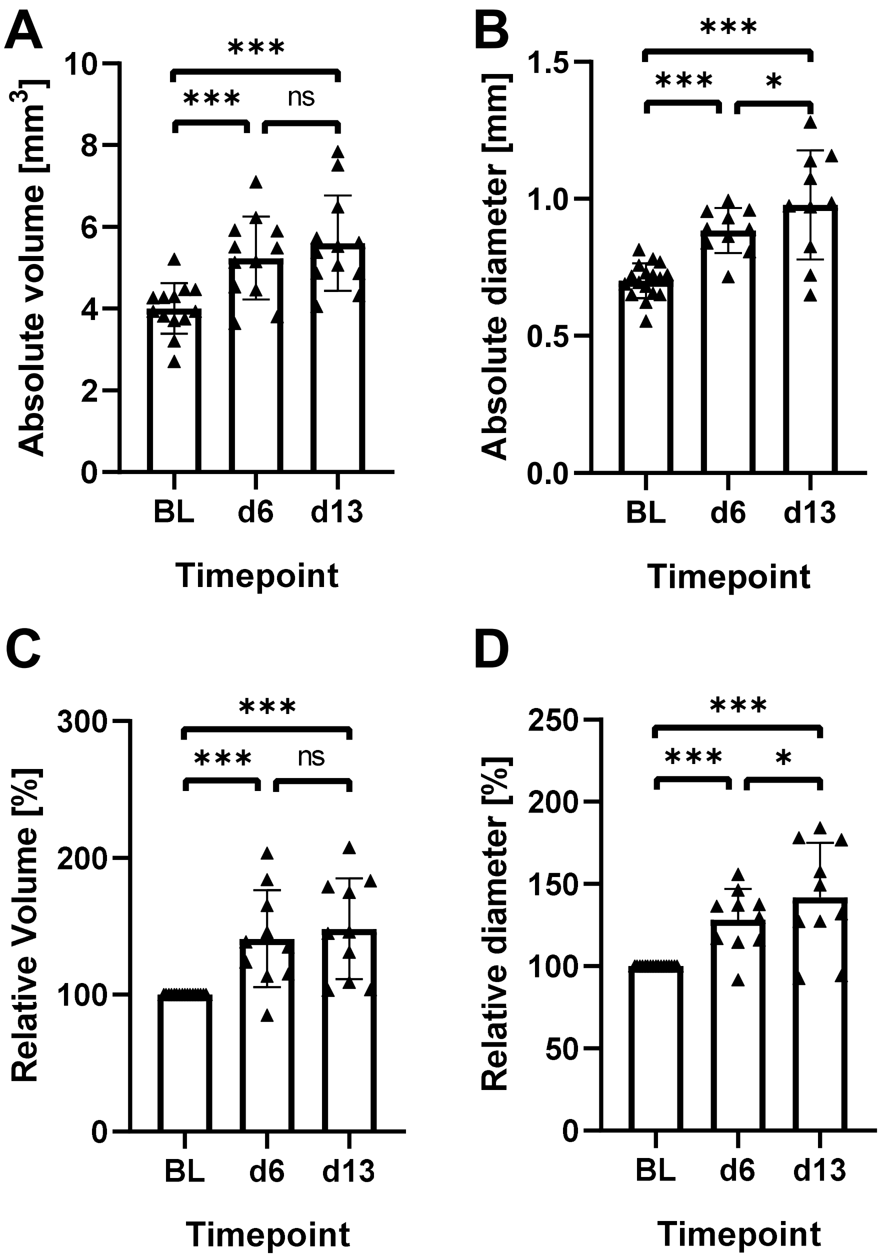

LMEM	P-value	Beta	Lower 95% CI	Upper 95% CI	Random effect
A	< 0.001	0.135	0.085	0.185	0.207
B	< 0.001	0.022	0.015	0.028	0.003
C	< 0.001	3.786	2.330	5.243	224.40
D	< 0.001	3.268	2.203	4.333	110.02

Suppl. Fig. 13

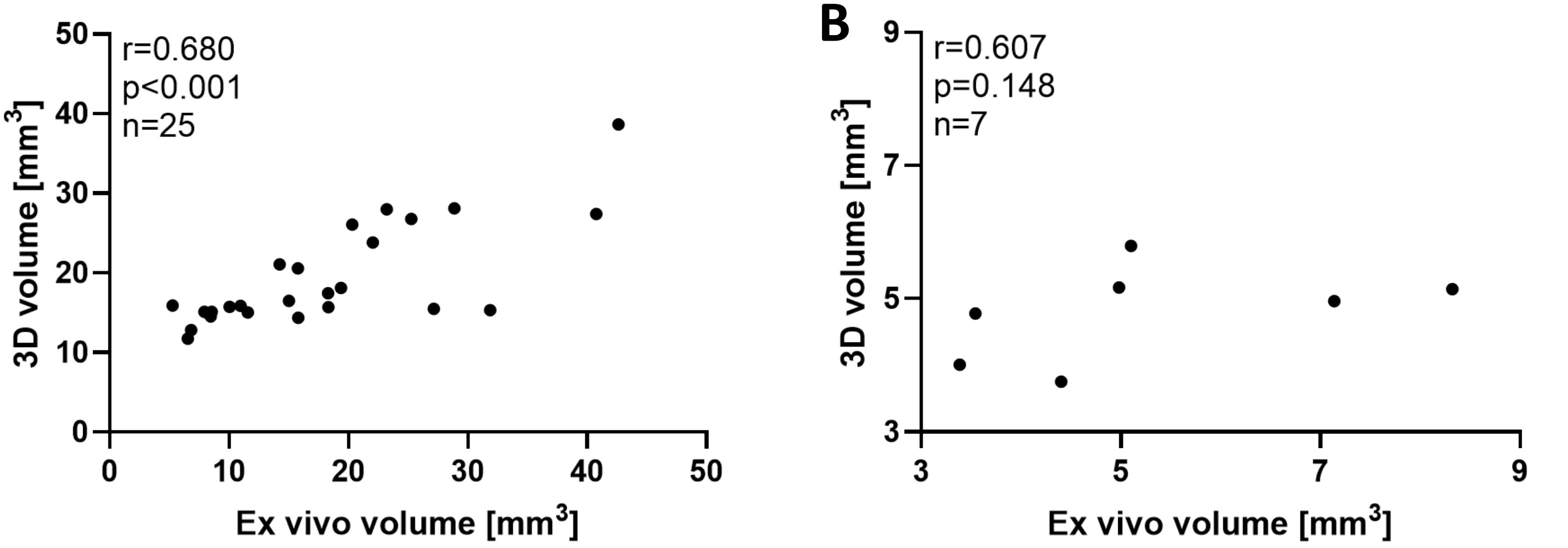
r=0.680
p<0.001
n=25
3D volume [mm3]
Ex vivo volume [mm3]
B
r=0.607
p=0.148
n=7
3D volume [mm3]
Ex vivo volume [mm3]
